# Antimicrobial Activity of Silver Diamine Fluoride and Sodium Fluoride Varnish Against Oral Bacteria: An In Vitro Comparative Study

**DOI:** 10.7759/cureus.106586

**Published:** 2026-04-07

**Authors:** Mannaa K Aldowsari, Mohammed H Alshamrani, Abdulmohsen H Alanazi, Faisal J Alsiwat, Salman A Almdani, Mohammed M AlRawaf, Waad E Alsaadi, Reem A Alajlan, Amjad M Alabdulmohsen

**Affiliations:** 1 Department of Pediatric Dentistry and Orthodontics, College of Dentistry, King Saud University, Riyadh, SAU; 2 Department of Dentistry, King Saud University, Riyadh, SAU

**Keywords:** agar disc diffusion, antimicrobial activity, lactobacillus acidophilus, porphyromonas gingivalis, silver diamine fluoride, sodium fluoride varnish, staphylococcus aureus, streptococcus mutans

## Abstract

Background and aim

Silver diamine fluoride (SDF) and sodium fluoride varnishes are used in caries management, but few studies have compared their antimicrobial activity head-to-head under standardized conditions. The present study aimed to compare the antimicrobial activity of two commercial silver diamine fluoride (SDF) formulations (Advantage Arrest and Riva Star Aqua), two 5% sodium fluoride varnishes (NovaBrighte and 3M ESPE Vanish), and two SDF-varnish combinations (Riva Star Aqua + 3M ESPE Vanish and Advantage Arrest + NovaBrighte) against *Streptococcus mutans, Porphyromonas gingivalis, Staphylococcus aureus*, and *Lactobacillus acidophilus* using an agar disk diffusion assay, with antimicrobial activity quantified by measurement of zones of inhibition. The null hypothesis was that there would be no statistically significant differences in antimicrobial activity among the tested agents or between individual and combination therapies.

Methods

Standardized agar disk diffusion assays were performed in three independent trials. Seven groups were tested as follows: untreated control, Riva Star Aqua, Advantage Arrest, 3M ESPE Vanish, NovaBrighte, Riva Star Aqua+3M ESPE Vanish, and Advantage Arrest+NovaBrighte. Per-species one-way ANOVA with Tukey post hoc tests and independent-samples t-tests were used to compare inhibition zones among the treatment groups and for pre-specified pairwise comparisons, respectively (α=0.05).

Results

All per-species ANOVA models were statistically significant (p<0.001). 3M ESPE Vanish produced no detectable inhibition zone against any species. The Advantage Arrest+NovaBrighte combination produced the largest mean inhibition zones against *Streptococcus mutans* (30.33±6.66 mm), *Porphyromonas gingivalis* (16.00±0.00 mm), *Staphylococcus aureus* (16.67±0.58 mm), and *Lactobacillus acidophilus* (15.17±2.75 mm). SDF Advantage Arrest alone outperformed Riva Star Aqua against *S. mutans *(16.33 vs. 13.33 mm; p=0.003) and *S. aureus* (13.67 vs. 11.00 mm; p=0.001). Adding 3M ESPE Vanish to Riva Star Aqua did not significantly change zones for any species (p>0.05).

Conclusions

SDF Advantage Arrest, alone and combined with NovaBrighte, showed the strongest antimicrobial effect. 3M ESPE Vanish had no direct antibacterial activity, while NovaBrighte did, highlighting formulation-dependent differences among sodium fluoride (NaF) varnishes.

## Introduction

Dental caries is the most common chronic oral disease and affects almost all age groups, though the burden is disproportionately high in children and communities with limited access to dental services [1-3]. The disease develops when the biofilm ecology shifts toward acidogenic and aciduric organisms, mainly *Streptococcus mutans* and Lactobacillus spp., which produce acids that progressively demineralize enamel and dentin [4,5]. Controlling or reducing these cariogenic bacteria is therefore a key objective in caries prevention [6-8].

Sodium fluoride varnishes (5%, 22,600 ppm F) have long been used in professional caries prevention owing to their ease of application, safety profile, and capacity to promote enamel remineralization [9,10]. Their mode of action, however, is primarily physicochemical; they modify the tooth surface but offer limited direct bactericidal activity, and once a lesion has cavitated, the antibacterial contribution of a conventional varnish is minimal [9]. Whether a given varnish formulation has measurable antibacterial activity depends on its specific excipients, as demonstrated by Jafari et al., who found that two of four tested varnishes produced no inhibition zones at all [11].

Silver diamine fluoride (SDF; 38% {Ag(NH₃)₂}F, 44,800 ppm F) combines the bactericidal properties of silver with the remineralizing action of fluoride [9,10]. It disrupts bacterial cell walls, inactivates metabolic enzymes, and penetrates dentin tubules, with antimicrobial activity that can persist after just one or two applications [12]. SDF has proven particularly useful in pediatric, geriatric, and community-based settings where conventional restorative care is hard to deliver. Co-application with potassium iodide has also helped address the well-known problem of black staining [13].

Despite the growing adoption of both SDF and fluoride varnishes, few studies have directly compared their antimicrobial efficacy across a panel of oral pathogens under standardized in vitro conditions [13-16]. Most published evidence focuses on clinical caries outcomes without isolating microbial effects, which limits our understanding of each agent's intrinsic antibacterial potential. It also remains unclear whether combining SDF with a fluoride varnish produces an additive antimicrobial benefit, and whether the choice of varnish matters in such combinations.

## Materials and methods

Study design and ethical considerations

This in vitro experimental study was conducted over three months at the Microbiology Laboratory, College of Dentistry, King Saud University (KSU), Riyadh, Saudi Arabia. As the study used commercially obtained reference bacterial strains and involved no human or animal subjects, formal ethical approval was not required.

Bacterial strains and culture conditions

Four reference bacterial strains were obtained from the American Type Culture Collection (ATCC; Manassas, VA) as follows: *Streptococcus mutans* (*S. mutans*) ATCC 25175, *Porphyromonas gingivalis* (*P. gingivalis*) ATCC 33277, *Staphylococcus aureus* (*S. aureus*) ATCC 25923, and *Lactobacillus acidophilus *(*L. acidophilus*) ATCC 4356. *S. aureus* ATCC 25923 was selected as the Clinical and Laboratory Standards Institute (CLSI) quality-control strain for antimicrobial susceptibility testing and to evaluate broad-spectrum efficacy against a facultative Gram-positive coccus found in the oral cavity of immunocompromised and denture-wearing patients.

Stock cultures were maintained at -80°C in brain-heart infusion (BHI) broth supplemented with 20% (v/v) glycerol. Working cultures were prepared by subculturing on appropriate solid media as follows: BHI agar for *S. mutans* (5% CO₂, 37°C, 24 h) and *S. aureus* (aerobic, 37°C, 24 h); de Man-Rogosa-Sharpe (MRS) agar for *L. acidophilus* (microaerophilic, 37°C, 48 h); and Brucella blood agar supplemented with hemin (5 µg/mL), vitamin K₁ (1 µg/mL), and 5% defibrinated sheep blood for *P. gingivalis* (anaerobic: 80% N₂, 10% CO₂, 10% H₂; 37°C, 72 h). Overnight broth cultures were standardized to a McFarland turbidity of 0.5 (≈1.5×10⁸ CFU/mL) using a densitometer.

Test materials and experimental groups

Seven treatment groups were tested against each of the four species, yielding 28 agent-species combinations as follows: group A - untreated control (sterile saline-loaded disk), group B - SDF Riva Star Aqua (Bayswater, Victoria: SDI Limited; 38% silver diamine fluoride), group C - SDF Advantage Arrest (West Palm Beach, FL: Elevate Oral Care; 38% silver diamine fluoride), group D - 3M ESPE Vanish 5% sodium fluoride white varnish with tri-calcium phosphate (St. Paul, MN: 3M/Solventum), group E - NovaBrighte 5% sodium fluoride varnish (Columbia, MO: Nanova Biomaterials Inc.), group F - combination: SDF Riva Star Aqua applied to the disk surface followed by 3M ESPE Vanish varnish, and group G - combination: SDF Advantage Arrest applied to the disk surface followed by NovaBrighte varnish.

Agar disk diffusion assay

Antimicrobial activity was assessed using the agar disk diffusion method. Mueller-Hinton agar plates (supplemented with 5% defibrinated sheep blood, hemin, and vitamin K₁ for *P. gingivalis*) were uniformly inoculated by spreading 100 µL of the standardized suspension across the surface with a sterile cotton swab. Sterile blank paper disks (6 mm diameter; Whatman No. 1) were loaded with 20 µL of the respective agent and placed equidistantly on the inoculated plates. After incubation under strain-specific conditions (section 2.2), zones of inhibition were measured as the full diameter (including the 6 mm disk) in millimeters using a digital caliper (resolution 0.01 mm). Three independent trials were conducted on separate occasions using freshly prepared cultures and reagents, yielding three measurements per agent-species combination.

Statistical analysis

For each bacterial species separately, the mean zone of inhibition (±standard deviation) was calculated from three independent trial measurements (n=3). Per-species one-way analysis of variance (ANOVA) was used to compare inhibition zones among the seven treatment groups; where ANOVA indicated a significant overall effect, Tukey's HSD post hoc test was applied. Pre-specified pairwise comparisons were performed using independent-samples t-tests as follows: (a) Riva Star Aqua vs. Advantage Arrest, (b) Advantage Arrest alone vs. Advantage Arrest+NovaBrighte, and (c) Riva Star Aqua alone vs. Riva Star Aqua+3M ESPE Vanish. A two-tailed p≤0.05 was considered statistically significant. Analyses were performed using IBM SPSS Statistics version 23.0 (Armonk, NY: IBM Corp.).

## Results

Zones of inhibition by bacterial species

Table 1 presents the mean zones of inhibition (±SD) for each treatment group against the four species. The untreated control and 3M ESPE Vanish produced no detectable inhibition for any species. Per-species ANOVA revealed significant differences among groups for the following four species: *S. mutans* (F {6,14}=34.90, p<0.001), *P. gingivalis* (F {6,14}=278.10, p<0.001), *S. aureus* (F {6,14}=339.85, p<0.001), and *L. acidophilus* (F {6,14}=50.21, p<0.001).

**Table 1:** Mean zones of inhibition by treatment group and bacterial species. *All p<0.001. Values represent mean±SD from three independent trials (n=3). RSA: Riva Star Aqua; AA: Advantage Arrest; 3M ESPE: 3M ESPE Vanish; NB: NovaBrighte; RSA+3M: Riva Star Aqua+3M ESPE Vanish; AA+NB: Advantage Arrest+NovaBrighte

Species	Control (A)	RSA (B)	AA (C)	3M ESPE (D)	NB (E)	RSA+3M (F)	AA+NB (G)	F (6,14)
S. mutans	0.00±0.00	13.33±0.58	16.33±0.58	0.00±0.00	19.00±3.61	17.00±3.46	30.33±6.66	34.90*
P. gingivalis	0.00±0.00	12.00±1.00	14.00±1.00	0.00±0.00	13.00±1.00	11.33±0.58	16.00±0.00	278.10*
S. aureus	0.00±0.00	11.00±0.00	13.67±0.58	0.00±0.00	13.17±1.04	11.00±1.00	16.67±0.58	339.85*
L. acidophilus	0.00±0.00	10.33±2.08	13.67±0.58	0.00±0.00	11.33±1.15	9.67±1.53	15.17±2.75	50.21*

The Advantage Arrest+NovaBrighte combination yielded the largest zones across all species, especially against *S. mutans* (30.33±6.66 mm). SDF Advantage Arrest alone consistently outperformed Riva Star Aqua. NovaBrighte varnish showed moderate antimicrobial activity as a single agent, with mean zones from 11.33 mm (*L. acidophilus*) to 19.00 mm (*S. mutans*). In contrast, 3M ESPE Vanish showed no antibacterial activity in any trial against any species, with all zones recorded as 0.00 mm.

Pairwise comparison of SDF formulations

Table 2 presents per-species pairwise comparisons between the two SDF formulations. Advantage Arrest produced significantly larger zones than Riva Star Aqua against *S. mutans* (p=0.003) and *S. aureus* (p=0.001). Differences against *P. gingivalis* (p=0.070) and *L. acidophilus *(p=0.056) showed a consistent trend favoring Advantage Arrest but did not reach significance at α=0.05.

**Table 2:** Pairwise comparison of SDF formulations: Riva Star Aqua vs. Advantage Arrest. *Significant values. Independent-samples t-test, n=3 per group. SDF: silver diamine fluoride

Species	Riva Star Aqua	Advantage Arrest	t	df	p-Value
S. mutans	13.33±0.58	16.33±0.58	-6.36	4	0.003*
P. gingivalis	12.00±1.00	14.00±1.00	-2.45	4	0.070
S. aureus	11.00±0.00	13.67±0.58	-8.00	4	0.001*
L. acidophilus	10.33±2.08	13.67±0.58	-2.67	4	0.056

Advantage Arrest alone vs. the combination

Table 3 compares Advantage Arrest alone with the Advantage Arrest+NovaBrighte combination. The combination produced significantly larger zones against *S. mutans* (p=0.022), *P. gingivalis* (p=0.026), and *S. aureus* (p=0.003). For *L. acidophilus*, the combination (15.17 mm) exceeded Advantage Arrest alone (13.67 mm), but the difference did not reach significance (p=0.408).

**Table 3:** Advantage Arrest alone vs. Advantage Arrest+NovaBrighte combination. *Significant values. Independent-samples t-test, n=3 per group.

Species	Advantage Arrest	AA+NovaBrighte	t	df	p-Value
S. mutans	16.33±0.58	30.33±6.66	-3.63	4	0.022*
P. gingivalis	14.00±1.00	16.00±0.00	-3.46	4	0.026*
S. aureus	13.67±0.58	16.67±0.58	-6.36	4	0.003*
L. acidophilus	13.67±0.58	15.17±2.75	-0.92	4	0.408

Riva Star Aqua alone vs. Riva Star Aqua+3M ESPE Vanish

Adding 3M ESPE Vanish to Riva Star Aqua did not significantly change the inhibition zones for any species. Mean zones were similar or slightly lower in the combination group as follows: *S. mutans* (17.00 vs. 13.33 mm; p=0.145), *P. gingivalis* (11.33 vs. 12.00 mm; p=0.374), *S. aureus* (11.00 vs. 11.00 mm; p=1.000), and *L. acidophilus* (9.67 vs. 10.33 mm; p=0.678). None of these comparisons reached significance.

3M ESPE Vanish and NovaBrighte: varnish comparison

3M ESPE Vanish produced no detectable zone of inhibition (0.00 mm) against any of the four species in all three trials. NovaBrighte, in contrast, showed mean zones of 19.00 mm (*S. mutans*), 13.00 mm (*P. gingivalis*), 13.17 mm (*S. aureus*), and 11.33 mm (*L. acidophilus*). This represents a clear qualitative difference between the two 5% sodium fluoride (NaF) varnish formulations.

## Discussion

This in vitro study compared the antimicrobial activity of two SDF formulations, two sodium fluoride varnishes, and two SDF-varnish combinations against four oral bacterial species using the agar disk diffusion method. The main findings were that SDF Advantage Arrest consistently produced larger inhibition zones than Riva Star Aqua; the Advantage Arrest+NovaBrighte combination yielded the greatest overall antimicrobial effect; NovaBrighte showed measurable single-agent activity; and 3M ESPE Vanish had no detectable antibacterial effect. The null hypothesis was rejected for most comparisons.

That Advantage Arrest outperformed Riva Star Aqua is noteworthy because both products share the same nominal SDF concentration (38%). This result suggests that differences in formulation excipients, pH, or silver ion bioavailability underlie the differential efficacy. Crystal and Niederman noted that compositional variations among commercial SDF products influence ion-release kinetics and clinical outcomes, and the present microbiological results support this observation [17]. The statistically significant differences against *S. mutans* (p=0.003) and *S. aureus* (p=0.001) reinforce the conclusion that SDF products should not be considered interchangeable.

The Advantage Arrest+NovaBrighte combination produced mean zones of 30.33 mm against *S. mutans*, significantly exceeding Advantage Arrest alone (p=0.022). One possible explanation is that the varnish prolongs silver ion contact with the bacterial inoculum, which may increase bactericidal activity. However, the progressive increase in zones across successive trials (23.0, 32.0, and 36.0 mm) contributed to high within-group variability (SD=6.66 mm). While this trend could reflect genuine inter-trial variation, it may also indicate a methodological factor such as differences in agent loading or culture preparation across trials. Future studies should standardize application volumes rigorously and increase replicate numbers. The term "synergy" is deliberately avoided, as formal synergy testing (fractional inhibitory concentration {FIC} index, checkerboard assay) was not performed; the observed effect is more accurately described as potentially additive pending confirmatory testing.

In contrast, the Riva Star Aqua+3M ESPE Vanish combination did not produce zones significantly different from Riva Star Aqua alone for any species (p=0.145-1.000). This is consistent with the fact that 3M ESPE Vanish itself had no measurable antibacterial activity. Adding an antimicrobially inactive varnish to SDF would not be expected to enhance bacterial inhibition. The marginal increase in zones for *S. mutans *(17.00 vs. 13.33 mm, p=0.145) was not significant and was accompanied by high variability (SD=3.46 mm). Together, these two combinations of results suggest that the choice of varnish matters as follows: pairing SDF with an antibacterially active varnish (NovaBrighte) produced a measurable benefit, while pairing with an inactive one (3M ESPE Vanish) did not.

Zhao et al. described the dual mechanism of SDF whereby silver ions disrupt bacterial cell membranes and denature glycolytic enzymes while fluoride inhibits enolase, together suppressing acid production [14,18,19]. Our results fit this broad-spectrum pattern as follows: SDF-containing groups showed activity against Gram-positive cariogenic species (*S. mutans, L. acidophilus*) and the Gram-negative obligate anaerobe *P. gingivalis*. Activity against *S. aureus* is clinically relevant given its presence in the oral cavity of patients with prosthetic devices or compromised immunity. Qazi et al. reported effective caries arrest by SDF in a pediatric population, and Ammar et al. showed that SDF outperformed nano-silver fluoride in reducing caries activity in primary teeth, lending clinical support to these in vitro findings [6,12]. Similar activity against cariogenic species was reported by Alvarez-Marín et al. in a recent disk diffusion study [19,20], and Klanliang et al. confirmed SDF-mediated biofilm disruption using an in situ model [21]. Sharma et al. reported comparable disk diffusion results when testing SDF against monospecies biofilms [22].

The absence of any inhibition zone around 3M ESPE Vanish disks warrants specific comment. This product is a 5% NaF varnish containing tri-calcium phosphate (TCP) and xylitol at concentrations intended to promote remineralization, not to kill bacteria. The manufacturer's literature makes no antibacterial claims for this product. Our finding is consistent with the study by Jafari et al., who tested four fluoride varnishes by disk diffusion and found that two of them (V-varnish and Preventa) produced no inhibition zones against *S. mutans*, while only varnishes containing higher concentrations of xylitol or CPP-ACP showed activity [17]. Erdem et al. similarly reported that Bifluorid 12, despite having the highest fluoride concentration among the varnishes they tested, produced the greatest number of viable *S. mutans* and *S. sobrinus* cells, indicating that high fluoride content alone does not predict antibacterial activity in the disk diffusion format [23]. The lack of activity may also reflect poor diffusion of the resin-based varnish through agar, a well-recognized limitation of the disk diffusion method for viscous or hydrophobic materials. Regardless of the cause, the result demonstrates that 5% NaF varnishes are not interchangeable in terms of antibacterial performance, and clinicians should not assume all varnishes have equivalent effects beyond remineralization.

NovaBrighte, despite also being a 5% NaF varnish, showed clear antibacterial activity across all four species. The zones (11.33-19.00 mm) place it in the moderate-to-strong range. This sets it apart from conventional sodium fluoride varnishes, which act mainly through remineralization rather than direct bacterial killing [9,17]. Its proprietary formulation, potentially incorporating bioactive carriers, likely accounts for this activity, consistent with reports that enriched varnish formulations containing calcium phosphate or xylitol can confer antimicrobial benefits beyond remineralization [17,24].

Strengths and clinical implications

This study tested four bacterial species with distinct roles in oral pathology, giving a wider antimicrobial profile than single-species designs. The inclusion of two varnishes with contrasting results (NovaBrighte active, 3M ESPE inactive) and two SDF-varnish combinations adds clinical relevance that single-product studies cannot provide. Three independent trials conducted on separate occasions strengthen reliability. All per-species ANOVA models reached high significance (p<0.001), confirming meaningful between-group differences. Our data support SDF-based strategies, particularly the Advantage Arrest+NovaBrighte combination, as non-invasive interventions for high-caries-risk populations in community settings with limited operative dental access.

This study has several limitations that should be kept in mind when interpreting the results. The agar disk diffusion assay, while widely used and reproducible, tests planktonic bacteria on an agar surface - a setting that does not capture salivary flow, pellicle formation, or the polymicrobial biofilm communities found in the mouth. We also tested only four species as monocultures; mixed-species biofilm models would give a more realistic picture of how these agents perform in vivo. Another concern is that viscous or resin-based products, such as 3M ESPE Vanish and NovaBrighte, may not diffuse through agar as readily as aqueous solutions, which could lead to smaller zones that underrepresent true activity. This is particularly relevant for the 3M ESPE Vanish result as follows: the zero zones may partly reflect poor agar diffusibility rather than complete absence of antibacterial potential, though the contrast with NovaBrighte (which is also a varnish yet produced clear zones) argues against diffusion as the sole explanation. With only three trials per group, statistical power was limited, especially for comparisons where effect sizes were small. We also did not include a positive antimicrobial control, such as chlorhexidine, which would have provided a useful benchmark. To build on the present findings, studies using multi-species biofilm models, time-kill assays, pH cycling, and formal synergy testing (checkerboard or FIC methods) are needed. Clinical trials in pediatric and geriatric populations will be required to determine whether the differences observed on agar plates hold up in practice.

## Conclusions

Within the limitations of this in vitro study, the findings suggest that SDF Advantage Arrest has greater antimicrobial activity than Riva Star Aqua against *S. mutans* and *S. aureus*, with a consistent trend of superiority across all four tested species. The results also indicate that NovaBrighte 5% sodium fluoride varnish has measurable antimicrobial activity as a single agent, whereas 3M ESPE Vanish 5% NaF varnish with TCP did not produce detectable inhibition zones under the present test conditions, highlighting formulation-dependent differences among NaF varnishes. In addition, the Advantage Arrest+NovaBrighte combination produced the largest inhibition zones across all tested species, with significant enhancement against *S. mutans, P. gingivalis*, and *S. aureus*, while adding 3M ESPE Vanish to Riva Star Aqua did not significantly improve inhibition zones. These findings suggest that SDF-based combination strategies merit further investigation for caries prevention; however, validation in biofilm models and clinical studies is required before clinical implications can be established.
